# Double trouble with double-booking: limitations and dangers of overlapping surgery

**DOI:** 10.1093/bjs/znac244

**Published:** 2022-07-15

**Authors:** Jaideep J Pandit, Satya Krishna Ramachandran, Meghana Pandit

**Affiliations:** Nuffield Department of Anaesthetics, Oxford University Hospitals NHS Foundation Trust, Oxford, UK; Department of Anesthesia, Beth Israel Deaconess Medical Center, Boston, MA, USA; Office of the Chief Medical Officer, Oxford University Hospitals NHS Foundation Trust, Oxford, UK

An operating theatre schedule must be of some significance if it results in one of the world’s leading hospitals (Massachusetts General Hospital, Boston, part of Harvard University’s group) paying over US $33 million (Euro 32.39 million; 1:0.98) as settlements (without liability)^1^. The schedule in question is overlapping surgery (more pejoratively, double-booking): organizing surgical lists in parallel, with a single senior surgeon moving from one theatre to the other. It aims to eliminate turnover time (downtime or gap time) between successive cases, thereby maximizing touchtime (clinician–patient contact time)^2^. Overlapping surgery is a step beyond overlapping anaesthesia^3^, where an anaesthesia room is used for induction while surgery is completed in the adjoining theatre. In overlapping surgery, non-critical portions of operations are started by junior surgeons in a parallel theatre, while the senior surgeon completes the critical parts in the first patient, before joining this second patient, then to commence on the critical periods.

Unsurprisingly, definitions of non-critical *versus* critical are subjective and the lawsuits above were triggered in part by the US Medicare/Medicaid requirement for the senior surgeon to be present at critical periods; if not, then overlapping surgery could be interpreted as billing fraud^1^. Concurrent or simultaneous operations are extensions of overlapping surgery, in which the senior surgeon is involved in two operations at once, leaving even for parts of the critical periods. National surgical societies that have examined the issue have criticized these latter practices^4^, notwithstanding the difficulties of precise definitions. There arise important issues of patient consent^5^: what is the proper information to convey to a patient when overlapping surgery is planned? What are the mitigations against risks that include, as identified in a US Senate report^6^, surgeons dividing their attention across two (or more) operating rooms for hours, being unable to return to the primary operation even when called, or failing to arrive on time leading to prolonged anaesthesia? These challenges are even more important in vulnerable patient groups, such as children^7^. Issues related to training have also been discussed elsewhere^8^; overlapping surgery requires trainees of sufficient seniority to commence non-critical portions of surgery as well as not being adversely affected by missing out on being present for critical parts. Understandably, safety concerns have been raised, with some studies reporting adverse outcomes^9^, but others not^10^.

With the size of waiting lists in the National Health Service (NHS) now exceeding 6 million patients, it is possible that overlapping surgery is a form of scheduling that could be viewed as maximizing productivity^11^. Versions of it are already being trialled in some NHS hospitals as high-intensity lists^12^. However, it is self-evident that overlapping surgery cannot, even in theory, increase the number of cases completed on a list.

Imagine a simple example of two parallel lists scheduled for 8 h (09.00 to 17.00 hours), and the operation in question has a duration of 90 min (including anaesthesia and positioning time) (*Fig. S1*). There is a fixed turnover time of 30 min between cases (used for theatre cleaning, mandatory paperwork, and equipment preparation, as well as ensuring minimum air changes). With a timely start, only four cases can be completed on each list (leaving a modest under-run of 30 min), which means eight cases in total for the two parallel lists. In an overlapping model, each case can be started 60 min into the ongoing case. Within each theatre, the turnover time remains at 30 min, fixed as it is by the essential duties described above. It is evident that there is no gain at all in overall productivity: a total of eight cases is still completed. One theatre finishes 30 min earlier than scheduled as before, but one theatre now finishes 30 min later. Not only is it impossible algebraically to describe an overlapping schedule that is more productive than two well planned independent lists, Morris *et al*.^13^ presented several theoretical schedules that resemble *Fig. S1b*, showing that, in many instances, overlapping surgery will result in *fewer* cases than could be completed in two independent theatres.

Observational studies of real-world lists have confirmed this productivity loss with overlapping surgery (*Table 1*)^14–16^. The reasons why overlapping surgery is, despite this, celebrated as a success are that authors erroneously focus on the gains made by a single surgeon, rather than viewing total productivity across two operating theatres; and place undue emphasis on the value gained by the seamless, uninterrupted working of the senior surgeon in the overlapping model. This last point may be important in systems such as those in the USA, where surgeons bill by hours of patient contact time, and their value is higher than that for other staff; maximizing their billable hours optimizes profits. Moreover, in systems like the US one, surgeons do not necessarily have a fixed weekly allocation of theatre space, but instead have variable allocations based on the work they are able to schedule. Overlapping surgery offers a means of increasing the satisfaction of busier/profitable surgeons. However, as might be expected because anaesthesia is induced in advance of the critical portions of surgery, both total duration of operation and, therefore, costs per case are *increased* by overlapping surgery (by up US $10 000 (Euro 9 820; 1:0.98 per list^15^). In a fee-for-service or payment-by-results system^17^, these costs might be offset by the additional income gained from performing some additional cases (*Table 1*), but this will not be the case as the NHS moves to block-funding contracts. Cost savings are possible as fewer senior surgeons are needed; however, decimating the surgical workforce is rarely mentioned as a driver for overlapping surgery.

**Table 1:** Data from three studies examining productivity (number of cases completed) with overlapping scheduling models

Reference	No. of cases in a list*	Expected no. of cases across two lists†	No. of cases with overlapping surgery‡	Loss of productivity with overlapping surgery (%)§
**Padegimas *et al*.^14^**	3	6	5	17
**Zachwieja *et al*.^15^**	7	14	8	43
**Murphy *et al*.^16^**	4	8	5	37

* Mean number of cases per list. †Expected number of cases across two conventionally scheduled parallel lists (twice the value in column 2). *Reported mean number of cases completed with overlapping surgery, across the 2 parallel theatres. §Productivity loss (the difference between the preceding two columns, expressed as a percentage).

Overlapping surgery will have little value in certain scenarios, but may be worthwhile in others. It cannot improve performance in a system where turnover times are already low or zero; where the entirety of the operation is deemed critical; or in very long operation where only a single patient can occupy the entire list. Conversely, it has potential in situations where turnover times are long^18^, where critical portions of surgery form about half of the duration of operation^18^, and for operations lasting less than 2 h^19^. Whether it is, therefore, worth considering in high-volume, low-complexity surgery models will depend on finding a solution to scheduling overlapping lists optimally. Current best practice is to schedule lists using the mean and variance of known operating times^20^. However, this does not help determine the optimal time to start the parallel, overlapping operation, and the research has not been done. Another opportunity for overlapping schedules is in NHS weekend, waiting list initiatives, where only one surgeon has volunteered for duty, or in staff-constrained specialties that nevertheless need to maintain surgical capacity; in the latter situation, as a temporary measure, overlapping operations could help maintain the service until recruitment has been achieved.

In conclusion, overlapping surgery should be viewed with great caution. It is not a means to increase the productivity of teams or theatre suites against the persistent and growing backlog of surgical waiting lists^21^, although it may increase the intensity of work for the primary surgeon. To avoid the double trouble of such double-booking, careful consideration of all the ethical and training concerns is required, the prior involvement and agreement of all stakeholders, and continued monitoring of financial, performance, and safety outcomes.

## Supplementary Material

znac244_Supplementary_Data
