# Supplementary figures and images for: Double trouble with double-booking: limitations and dangers of overlapping surgery
Source: Br J Surg. 2022 Jul 15;109(9):787–9. doi: 10.1093/bjs/znac244 (PMC10364735; doi:10.1093/bjs/znac244)

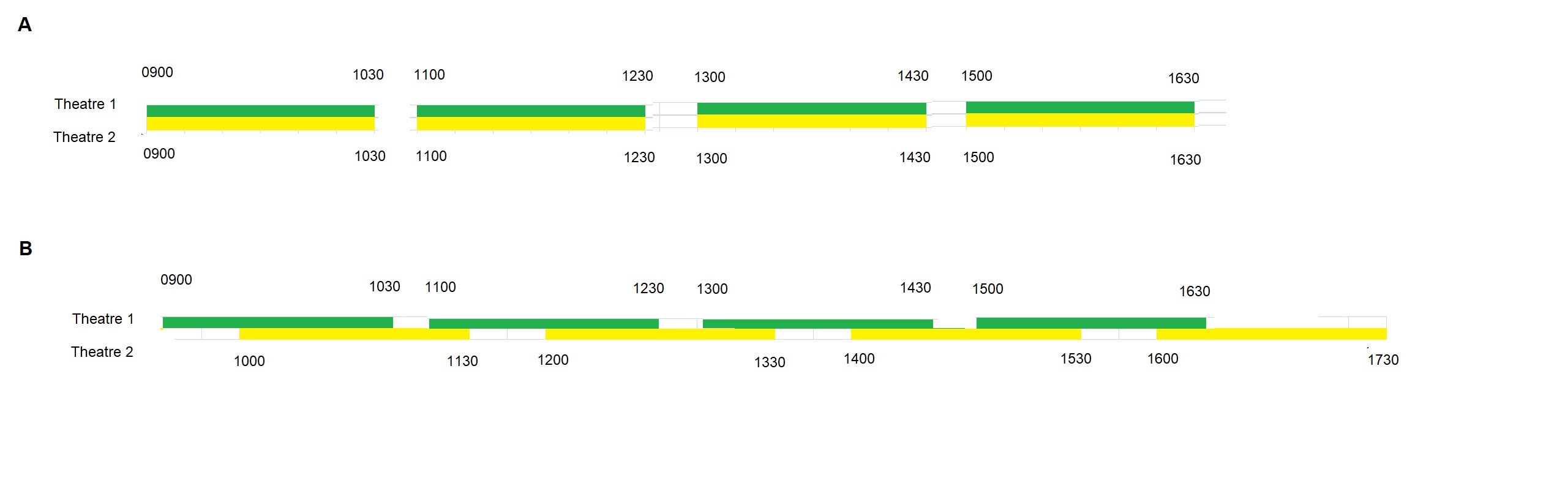

Supplement: znac244_Supplementary_Data [file znac244_supplementary_data.jpeg]
